# Posterior Wall Isolation in Atrial Fibrillation Ablation

**DOI:** 10.19102/icrm.2018.090602

**Published:** 2018-06-15

**Authors:** Khola S. Tahir, J. Paul Mounsey, James P. Hummel

**Affiliations:** ^1^Department of Internal Medicine, Division of Cardiology, University of North Carolina at Chapel Hill, NC, USA; ^2^Department of Internal Medicine, Division of Cardiac Electrophysiology, University of North Carolina at Chapel Hill, NC, USA

**Keywords:** Atrial fibrillation, catheter ablation, posterior wall isolation

## Abstract

Catheter ablation is widely utilized for the management of atrial fibrillation (AF), particularly in patients who are refractory to medical therapy. The left atrium appears to play a dominant role in the condition of most patients with AF and, in particular, the posterior wall and pulmonary veins frequently harbor sources of fibrillation. Currently, the role of posterior wall isolation during catheter ablation of AF is controversial. In this review, we will examine the mechanistic role of the posterior left atrium, discuss the technical challenges of ablating in the posterior wall and the evolution of strategies to achieve isolation with catheter approaches, and review the relevant literature to date.

## Introduction

Atrial fibrillation (AF) is the most common sustained arrhythmia in humans and is associated with significant morbidity and mortality. Furthermore, in recent years, the prevalence of AF has risen and is projected to increase threefold by 2050 as the population ages.^1^ Catheter ablation improves quality of life in patients with AF and has emerged as either a first-line therapy option or as an alternative therapy in those patients with symptomatic AF refractory to medical therapy. Notably, the left atrium (LA) appears to play a dominant role in the establishment and maintenance of AF in most patients with the condition and, in particular, the posterior wall and pulmonary veins (PVs) frequently harbor sources of fibrillation. In paroxysmal AF, the posterior wall has been demonstrated to have a role in both the initiation and maintenance of AF episodes. Ectopic beats originating from the PVs initiate the majority of paroxysmal AF,^2^ and frequency gradients emanating from the posterior LA to the rest of the atria are often seen during sustained fibrillation.^3^ PV isolation (PVI) has emerged as a highly effective therapy for patients with paroxysmal AF.^2,4^ However, PVI alone, which exudes a variable amount of posterior wall tissue, is often an insufficient treatment method for patients with persistent AF.^5^

Observations from surgical isolation of the posterior LA in humans have implicated the region between the PVs as a potential major contributor to persistent AF. The original Cox maze procedure and its modifications, which generally include complete exclusion of the posterior LA, have demonstrated efficacy even in those with longstanding persistent AF.^6,7^ During encircling of the posterior wall, the shortest atrial cycle lengths are often found in this region.^8^ When isolated en bloc in patients with persistent AF, it has been found that the posterior LA can still sustain AF in some patients, while the remaining atria cannot.^9^ These observations have motivated the development of catheter-based techniques to achieve complete posterior wall isolation in an effort to improve ablation outcomes.

Currently, the role for posterior wall isolation during catheter ablation of AF is controversial. In this review, we will examine the mechanistic role of the posterior LA, discuss the technical challenges of ablating in the posterior wall and the evolution of strategies to achieve isolation with catheter approaches, and review the relevant literature available to date. Most studies show an incremental benefit of posterior wall ablation in addition to PVI in patients with persistent AF, although patient selection for this strategy remains an area of active investigation.

## The role of the posterior left atrium in atrial fibrillation

The posterior LA may play a critical role in the initiation and maintenance of AF for multiple reasons. First, the posterior wall and its myocardial extensions into the PVs share similar embryologic origins. Specialized conduction tissue derived from the heart tube with intrinsic pacemaker activity has been demonstrated in these myocardial sleeves.^10,11^ Anatomically, there is significant heterogeneity in terms of myocardial fiber orientation within the PV antra and posterior wall, creating anisotropic conduction favoring local reentry. Electrophysiologically, myocytes within the PVs and posterior wall have properties distinct from those of other sites in the atria, with larger late sodium currents, smaller inward rectifier potassium currents, reduced resting membrane potential, and larger intracellular calcium transient.^12,13^ The posterior LA and PV myocytes exhibit shorter action potential durations and slower phase 0 upstroke velocities.^13^ The posterior wall also houses the main autonomic ganglionic plexi (GP), located within epicardial fat pads and the ligament of Marshall. Stimulation of the GP can result in both early afterdepolarization of the PVs or atrial myocytes and a shortening of the atrial action potential duration.

In addition to these baseline differences, elevated pressures may disproportionately affect the posterior LA to promote regional mechanisms for AF. Acutely, rapid activation in the PV region has been demonstrated in stretch-related AF, with the level of spatiotemporal organization correlated with the degree of pressure elevation.^14^ Over time, areas of the LA exposed to the highest wall stress, particularly the areas around the PV antrum and high posterior wall, have been correlated with low voltage and electrical scarring during mapping.^15^ Pathologic studies have similarly shown increased fibrosis in the posterior LA of patients with chronic AF and mitral valve disease.^16^

Recent evidence supports a major role for rotors as a driver for AF in both animals and humans. The electrophysiologic and anatomic conditions present in the posterior wall provide an ideal substrate for the formation and maintenance of rotors. Mandapati et al. found the posterior LA to be responsible for 80% of high-frequency rotors in an isolated sheep heart model.^17^ Favorable conditions for rotor formation include (1) triggers from PVs; (2) anisotropy and abrupt changes in wall thickness in the posterior wall that create source–sink mismatch, leading to wavebreak/reentry; (3) shorter local refractory periods that allow high-frequency driving rotors to persist; and (4) fibrosis that serves as unidirectional block, which favors rotor formation and acts as an anchor for rotor maintenance. During clinical AF ablation procedures in humans, mapping often localizes stable rotors or focal sources^18^ as well as important complex fractionated electrograms^19^ in the LA posterior wall and roof.

The unique conditions that are present in the posterior LA thus support fibrillation by multiple mechanisms. Abnormal automaticity, triggered activity, and/or microreentry can serve as focal sources for AF. Favorable electrophysiologic properties, tissue anisotropy, and fibrosis provide an ideal substrate for wavebreak and rotor formation, which can perpetuate AF. The posterior wall also contains the main GP, and the stimulation of these can both trigger AF initiation and favor its maintenance. In certain patients, multiple mechanisms can exist simultaneously to further promote AF.

## Challenges involving posterior wall isolation

**Figure 1:**
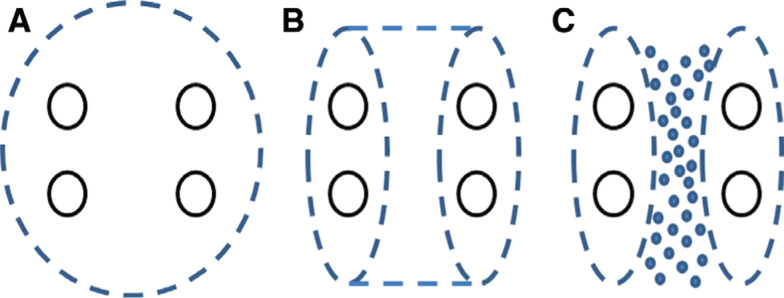
Lesion sets for LA posterior wall isolation. **A:** Single ring. **B:** Box lesion set. **C:** Obliteration of LA posterior wall potentials.

Complete isolation of the posterior wall can be technically challenging. The PV and posterior wall block isolated during a Cox maze III procedure encompasses roughly 35% of the LA endocardial surface area.^20^ Thus, isolation from the endocardial side requires long linear ablation lesions or extensive focal ablation of the posterior wall **(Figure 1)**. Improvements in radiofrequency catheter technology have made endocardial posterior wall isolation feasible. However, even with the use of irrigated catheters and force-sensing capabilities, the creation of durable lines of block when ablating in a point-by-point fashion from the endocardial surface using current technology remains difficult. Furthermore, attempts to improve lesion durability endocardially with increased contact force and power may increase procedural risks, including injury to periesophageal branches of the vagus nerve as well as esophageal injury such as the creation of atrioesophageal fistulae. Irrigated ablation catheters are now being widely used for ablation. There is an increased risk of atrioesophageal fistulae formation with the use of these catheters because they make deeper lesions and can more easily damage the esophageal tissue.

Luminal esophageal temperature monitoring with an esophageal temperature probe is used routinely in AF ablation procedures to help limit thermal injury to the esophagus from the ablation catheter. This esophageal probe needs to be readjusted throughout the procedure to ensure that it is in fact adjacent to the ablation site in order to detect the temperature accurately in the esophagus.^21^ Also, it is important to note that the esophageal temperature can continue to rise despite an interruption in ablation or a reduction in power, leading to esophageal injury. Retraction of the esophagus using an esophageal stylet is another strategy that can be employed to help decrease the incidence of esophageal thermal injuries. A randomized clinical trial comparing the esophageal stylet to no intervention and the impact on esophageal injury or erosion during AF ablation is currently underway.

Ablation from the epicardial surface allows for good anatomic placement of the catheter with direct visualization of linear lesions. In addition, the energy source is directed away from the esophagus, potentially decreasing the chance of injury. However, even under direct visualization, uniform catheter contact can be difficult due to the pericardial reflections from PV recesses and the oblique and transverse sinuses.^22^ Lesion transmurality can also be limited by excessive heating of the epicardial layers, resulting in increased tissue impedance, while the deeper endocardium is cooled by intracavitary blood flow. Thus, gaps in epicardial linear lesions during minimally invasive surgical approaches are also not uncommon. Muneretto et al. found 17% of patients to have gaps in their epicardial posterior box lesion set at 30 days after ablation.^23^ Such hybrid approaches, combining the best aspects of minimally invasive epicardial ablation and endocardial catheter ablation, are promising, but also reveal the challenges with achieving durable posterior wall isolation. Kumar et al. were able to demonstrate posterior wall isolation in roughly half of patients with combined epicardial–endocardial ablation.^24^ However, in five patients in whom posterior wall isolation was achieved at the index procedure who returned for subsequent repeat ablations, all had recurrence of conduction to the posterior wall.

## Differing criteria for isolation

**Figure 2:**
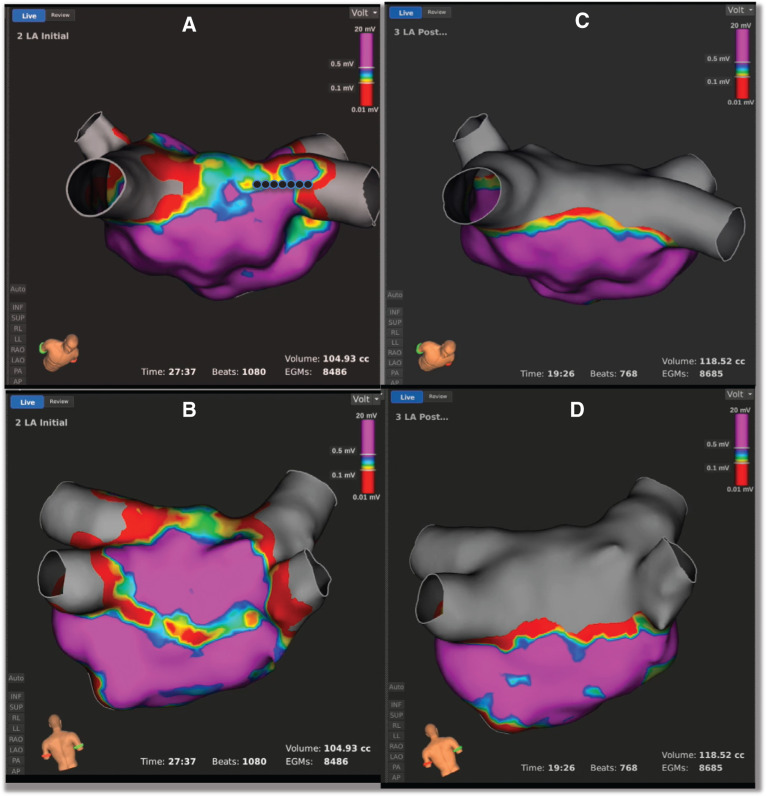
LA posterior wall voltage maps. **A and B:** Voltage maps of the LA acquired immediately after epicardial linear ablation targeted at LA posterior wall isolation. **C and D:** Voltage maps of the LA acquired after completion of the roof line with endocardial ablation (black dots) showing uniformly low LA posterior wall voltage. Note that the normal LA posterior wall voltage in **B** is reduced to < 0.1 mV after roof line completion **(D)**. The maps were acquired using the Rhythmia cardiac mapping hardware and software (Boston Scientific, Natick, MA, USA). Epicardial ablation was completed using the COBRA Fusion system (Atricure, Mason, OH, USA). Image reproduced courtesy of Dr. Andy C. Kiser and Dr. J. Paul Mounsey. LA: left atrial/atrium.

**Figure 3:**
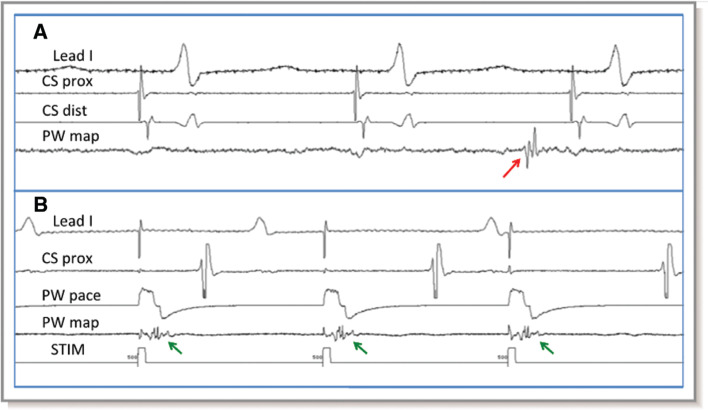
Posterior wall isolation—entrance and exit block. **A:** Note the dissociated potential on the posterior wall mapping catheter (posterior wall map, red arrow). **B:** Pacing from the posterior mapping catheter resulted in LA posterior wall capture dissociated from the underlying sinus rhythm (green arrows). LA: left atrial/atrium; CS prox: proximal coronary sinus; CS dist: distal coronary sinus; PW: posterior wall; STIM: simulus.

So, what does LA posterior wall isolation mean? Different groups have utilized different criteria for defining it. For example, several groups, using linear ablation lesions intended to isolate the LA posterior wall and PVs, searched for an absence of electrical activity on electroanatomic maps of the LA posterior wall (usually defined as voltage < 0.1 mV) **(Figure 2)** and an inability to capture outside the box during high-output pacing from within the box.^25–27^ Using similar ablation techniques, Sanders et al.^28^ and Tamborero et al.^29^ looked in addition for dissociation of LA posterior wall potentials originating from the isolated segment **(Figure 3)**. They also pointed out that evidence for LA posterior wall isolation was much stronger when the inability to conduct to the remaining atria with pacing from within a silent surrounded LA posterior wall was accompanied by observing the local capture in the proximal bipole of the pacing catheter when possible. Kumar et al. noted that, when these criteria were rigidly applied, that, although it was possible to achieve electrical silence of the posterior wall (< 0.1 mV) with the inability to capture the heart with pacing from within the LA box in essentially all cases, in a significant proportion of patients, LA posterior wall dissociation could not be achieved, and it was not always possible to demonstrate local LA capture with pacing from within the box (ie, evidence of LA posterior wall block was frequently uncertain—see also the following).^24^

The alternative technique for LA posterior wall isolation—the obliteration of LA posterior wall potentials with multiple ablation lesions^30,31^—results in electrical silence of the LA posterior wall in all cases. The outcome can only be defined by voltage mapping because there should be no electrically viable myocardium in the ablated segments to allow for reliable pace capture. Thus, there is absent near-field electrical activity in these cases, and only entrance block can be demonstrated.

It seems clear that low posterior wall voltage after ablation does not equate with LA posterior wall isolation. The appearance of a dissociated LA posterior wall potential in the ablated segment that can be captured with local pacing without conduction to the remainder of the atrium does define LA posterior wall isolation. In the absence of a dissociated potential, it is mandatory to demonstrate local LA posterior wall capture with exit block; the inability to pace the heart with local LA posterior wall stimulation does not define exit block in an apparently electrically silent LA posterior wall. When posterior wall ablation is done via obliteration of posterior wall potentials with multiple ablations, the efficacy can only be assessed as low posterior wall voltage.

## Ablation strategies for posterior wall isolation

Research has suggested that the exclusion of more posterior LA tissue around the PV antra results in better success rates when performing PVI.^5^ Owing to the success rates seen with surgical maze procedures and with the presence of multiple potential mechanisms for AF sources in the posterior wall, along with the challenges of achieving durable isolation of this area, several ablation strategies to completely isolate this region have evolved **(Figure 1)**.

### Single ring

All variants of the Cox maze procedure, including the Cox maze III procedure, involve isolating the posterior LA, generally as a single circle encompassing the PVs and posterior wall, and have been associated with favorable results.^6,7^ Early endocardial catheter attempts to replicate posterior wall isolation with a single ring **(Figure 1A)**, however, have been unsuccessful. Ernst et al. attempted isolation of the posterior wall in 13 patients with a 4-mm nonirrigated-tip catheter, and achieved isolation in none of them.^32^ Thomas et al. demonstrated the feasibility of this approach in 41 patients using an open-irrigated ablation catheter and deflectable sheath. Isolation of the posterior wall was achieved in 39 of the 41 patients, with a procedure time of 337 minutes including 176 minutes of fluoroscopy.^33^ Notably, however, they often had difficulty achieving block in the roof portion of the circle, requiring ablation be performed on the posterior wall to complete isolation. Two patients developed tamponade.^34^ Kumagai et al. reported on posterior wall isolation with a single ring using noncontact mapping and an 8-mm-tip ablation catheter in 188 patients.^25^ They achieved isolation in 92% of them, with a mean procedure and fluoroscopy time of 152 minutes and 31 minutes, respectively.^25^ During a mean follow-up period of nine months, success rates in paroxysmal, persistent, and longstanding persistent AF were 87%, 69%, and 42%, respectively. In patients with recurrence who were symptomatic, a second ablation procedure was performed, and all patients were found to have conduction gaps.^25^

One potential advantage of the single ring approach is that it may limit the exposure of the esophagus to radiofrequency energy, as long vertical posterior lines along the esophagus are replaced instead by a single horizontal line crossing the esophagus in this technique. However, the major disadvantage is that recurrent conduction is likely to occur at some point along the long perimeter, and such may compromise isolation of the PVs as well as the posterior wall.

### Pulmonary vein isolation plus box lesion set

Although PVI alone is insufficient in 20% to 60% of patients with persistent AF, it is still an effective therapy for a significant number of patients, including even those with longstanding persistent AF.^33^ Thus, it seems reasonable to strive for durable PVI prior to adding additional lesions. The PVI plus box lesion set uses double circles around the veins as anchors for posterior wall isolation, which is created by the addition of a roof line connecting the superior PVs and a low posterior line connecting the inferior veins **(Figure 1B)**.

Sanders et al. conducted a prospective clinical study in 27 patients with chronic (persistent for more than six months) AF, evaluating the feasibility and clinical outcomes of posterior LA isolation in addition to PVI.^28^ Successful posterior wall isolation, defined by an absence of local electrograms, was achieved in all patients, with dissociation of the posterior wall seen in only six. In three patients, sinus rhythm could not be maintained after completion of the lesion set and were thus considered failures requiring additional ablation. Twelve of the remaining 24 patients developed recurrent arrhythmias (specifically, atrial tachycardia in four and AF in eight). Among nine patients who returned for repeat ablation, the recurrence of conduction to the posterior wall was found in six. Over 23 months ± three months of follow-up, sinus rhythm was maintained off antiarrhythmic drugs in 12 patients (44%) following a single procedure; four additional patients (15%) maintained sinus rhythm after a second procedure to reisolate the posterior wall.

Chen et al. further investigated the addition of the posterior box to a PVI lesion set in 42 patients with paroxysmal (43%), persistent (33%), or permanent (24%) AF.^27^ If isolation was not achieved following the completion of the roof and low posterior lines, then residual signals in the posterior wall were targeted, with the endpoint being electrical silence and an inability to capture the posterior wall. After a mean follow-up of 20 months ± four months, 14 patients (33%) had recurrent arrhythmias (specifically, atrial tachycardia in four, AF in eight, and both in two). In six patients who underwent repeat ablation, all demonstrated recurrent conduction of the posterior wall. Clinical success rates were 94%, 86%, and 60% for paroxysmal, persistent, and permanent AF, respectively, although 18 patients remained on antiarrhythmic therapy.

Saad and Slater describe their experience with this lesion set in 25 patients with persistent or longstanding AF.^26^ In this study, the isolation and eradication of “dormant” conduction, identified with adenosine, was achieved in all individuals. Following 16 months ± two months of follow-up, arrhythmia occurred in 20% of patients, with all incidents consisting of perimitral flutter. Among these five patients who underwent redo ablation for atypical flutter, all had persisting intact isolation of the LA posterior wall. Kumar et al. report their results in 57 patients with significant structural heart disease, failed previous endocardial ablation, or longstanding persistent AF, of whom 30 underwent endocardial ablation and 27 underwent hybrid endocardial and epicardial ablation, respectively.^24^ Their endpoint, entrance and exit block with the ability to dissociate the posterior wall from the remaining atria, was difficult to achieve, with successful isolation of the posterior wall in only 23% of endocardial-only ablations and in 52% of hybrid ablations. Over a median follow-up of 10 months, recurrent arrhythmias occurred in 25 patients (44%) (specifically, atrial tachycardia in seven and AF in 18). Among five patients undergoing repeat ablation who had achieved isolation at the index procedure, all had recurrent conduction of the posterior wall. Kumar et al. also raise the question of the importance of achieving entrance and exit block, as patients with complete isolation did not have lower recurrence rates versus those without.^24^

### Obliteration of posterior wall potentials

From the findings of studies on posterior wall isolation by linear lesions, it became clear that it is difficult to create durable isolation of the posterior LA. In addition, it is also possible that complete isolation was not necessary to eradicate AF if enough ablation was performed through substrate-containing active sources (ie, “debulking” of the LA). Thus, another approach was born, in which, after PVI, extensive ablation is performed, targeting any signal in the posterior LA between the two antral circles **(Figure 2C)**. Rather than entrance and exit block of the posterior wall, this approach focuses on extensive ablation to address the potential rotors and GP present in this area, with the endpoint of elimination of all LA posterior wall electrograms and electrical silence.

Bai et al. describe their results with this approach in comparison with traditional antral PVI in a consecutive series of 52 patients with persistent AF.^31^ In their study, the first 20 patients underwent PV antral ablation and, in the next 32 patients, the PVI was extended to include obliteration of all LA posterior wall potentials. The assessment of durable block was vigorous, with all patients being brought back to the electrophysiology laboratory three months after any ablation to check for recurrent conduction, and to be reablated if necessary. Follow-up was not started until durable isolation had been achieved. Sixty percent of the PVI group and 63% of the PVI plus posterior wall group remained isolated at three months. Better clinical results were achieved in the posterior wall group with one-, two-, and three-year freedom from recurrence rates of 75%, 56%, and 44% versus 20%, 15%, and 10% in the PVI-only group. Of note, recurrences in the PVI group were mostly AF (85%) as compared with atrial tachycardia (69%) in the posterior wall group.

Segerson et al. described their initial experience with ablation of the posterior wall and septal walls of the LA in 2010.^30^ They conducted a single-center study involving 118 patients with persistent or longstanding AF undergoing AF ablation. They modified their usual AF ablation protocol to include debulking of the LA by isolating the posterior and septal walls and employed delayed-enhancement magnetic resonance imaging at three months’ postablation to look for scar formation. Following evaluation, they found the posterior wall scar burden present on magnetic resonance imaging to be the strongest predictor of a successful ablation; furthermore, 69.5% of the patients in this study had a successful ablation, defined as such by the absence of AF or atrial flutter lasting more than one minute. The complication rates were also low, with no occurrence of esophageal complications, and there was only one patient who demonstrated cardiac perforation and tamponade, which was managed with pericardiocentesis. This study is distinct from the others described herein, in that ablation was targeted to the septum as well as to the posterior wall. The authors concluded that early aggressive treatment of the posterior and septal walls of the LA, which are known to have a pathogenic substrate for AF, improves ablation outcomes.

## Randomized clinical trials

Results from randomized studies evaluating the benefits of posterior wall isolation have been conflicting **(Table 1)**. Kim et al. prospectively randomized 120 patients with persistent AF^35^ to two lesion sets—one with and one without isolation of the posterior wall. All patients underwent antral PVI, a roof line, and an anterior line to the mitral annulus. The treatment group also underwent an additional low posterior line with an endpoint of either dissociation or electrical silence of the posterior wall and the inability to capture with pacing. Notably, there was a higher rate of AF termination during the procedure (75% versus 66%) and a higher freedom from recurrent arrhythmias at one year (83% versus 63%) in the posterior wall group. No difference in LA function at one year was found between the two groups.

Additionally, Tamborero et al. found no benefit of additional posterior wall isolation in 120 patients with paroxysmal, persistent, or longstanding AF.^29^ Following circumferential PV ablation, patients were randomized to receive either an additional roof line or complete LA posterior wall isolation. Posterior wall isolation was defined as both entrance and exit block, and was achieved in 92% of the treatment group. There was no difference in outcome between the two randomized groups. However, nonparoxysmal AF patients constituted only 40% of the cohort, and the rate of freedom from AF (40%) was lower than that seen in similar studies of circumferential PVI alone in patients with predominantly paroxysmal AF. It is possible that, in a cohort containing more persistent AF patients, the potential benefit of LA posterior wall isolation might have become more apparent.

## Conclusions

PVI alone is often insufficient in patients with persistent AF. The posterior wall between the PVs contains multiple potential mechanisms that can initiate and sustain AF, and thus may be important in patients who fail or are likely to fail PV ablation alone. However, it can be technically challenging to isolate the entire posterior wall, and isolation often requires significant exposure of the esophagus to the ablation energy source. Combined endocardial–epicardial hybrid procedures may be helpful in achieving durable isolation. Unfortunately, there is a paucity of randomized clinical trials evaluating the outcomes of posterior wall isolation in patients with AF. More randomized trial data are clearly needed, and it is likely that posterior wall isolation for longstanding persistent AF will improve outcomes based on available and future data.

### Future directions

Although PVI alone is often insufficient in persistent and longstanding persistent AF, there remains an important minority of patients who do well with limited ablation. Better signal processing and imaging techniques are needed to identify these patients so that they are not accidentally exposed to the additional risks of full posterior wall isolation. In addition, better assessment of fibrosis and the identification of AF sources in the posterior wall may be helpful to improve patient selection for posterior wall isolation in those who do fail PVI. Improvements in ablation technology to allow real-time lesion assessment may aid in the creation of long durable linear lesions.

## Figures and Tables

**Table 1:** Randomized and Nonrandomized Series Evaluating Posterior Wall Isolation

Lesion Set	Study	Series Type	Number of Patients	Patient Age	Procedure Time	Fluoroscopy Time	Follow-up	Postprocedural Freedom from AF
PVI + box (endocardial)	Kumar et al.^24^	Nonrandomized	30	64 years	114 ± 35 minutes	65 ± 21 minutes	10 months	56.1%
PVI + box (hybrid)	Kumar et al.^24^	Nonrandomized	27	61 years	62 ± 19 minutes	63 ± 20 minutes	10 months	56.1%
Single ring	Kumagai et al.^25^	Nonrandomized	24*	–	152 ± 23 minutes	31 ± 18 minutes	9 ± 4 months	42.0%
	Ernst et al.^32^	Nonrandomized	13	60 years	474 ± 84 minutes	105 ± 22 minutes	0.84 months	0.0%
	Thomas et al.^33^	Nonrandomized	41	58 years	337 ± 76 minutes	176 ± 93 minutes	6.5 ± 2.3 months	87.8%
PVI + box	Saad and Slater^26^	Nonrandomized	25	65 years	40 ± 9 minutes	–	16 ± 2 months	80.0%
	Chen et al.^27^	Nonrandomized	42	56 years	261 ± 34 minutes	47 ± 11 minutes	20 ± 4 months	66.6%
	Sanders et al.^28^	Nonrandomized	27	57 years	199 ± 46 minutes	64 ± 16 minutes	23 ± 3 months	44.0%
	Segerson et al.^30^	Nonrandomized	118	68 years	240 ± 52 minutes	96 ± 26 minutes	12 months	69.5%
CPVA	Tamborero et al.^29^	Randomized	60	53 years	114 ± 23.4 minutes	23.2 ± 7.8 minutes	9.8 ± 4.3 months	55.0%
CPVA + LA posterior wall isolation	Tamborero et al.^29^	Randomized	60	53 years	120.9 ± 37.7 minutes	22.6 ± 8.3 minutes	9.8 ± 4.3 months	55.0%
Obliteration of all posterior wall electrograms	Bai et al.^31^	Nonrandomized	32	63 years	216 ± 66 minutes	62 ± 26 minutes	36 months	40.0%
PVI	Kim et al.^35^	Randomized	60	58 years	154.9 ± 57.1 minutes	–	12 months	81.6%
PVI + box	Kim et al.^35^	Randomized	60	57 years	163.1 ± 47.2 minutes	–	12 months	83.3%

AF: atrial fibrillation; PVI: pulmonary vein isolation; CPVA: circumferential pulmonary vein ablation; LA: left atrial/atrium.

*Persistent.
